# Purification and Characterization of a New Thermostable, Haloalkaline, Solvent Stable, and Detergent Compatible Serine Protease from* Geobacillus toebii* Strain LBT 77

**DOI:** 10.1155/2016/9178962

**Published:** 2016-03-16

**Authors:** Wajdi Thebti, Yosra Riahi, Omrane Belhadj

**Affiliations:** Laboratory of Biochemistry & Technobiology, Faculty of Science of Tunis, Tunis El Manar University, Farhat Hached University Campus, 2092 El Manar, Tunisia

## Abstract

A new thermostable, haloalkaline, solvent stable SDS-induced serine protease was purified and characterized from a thermophilic* Geobacillus toebii* LBT 77 newly isolated from a Tunisian hot spring. This study reveals the potential of the protease from* Geobacillus toebii* LBT 77 as an additive to detergent with spectacular proprieties described for the first time. The protease was purified to homogeneity by ammonium sulfate precipitation followed by Sephadex G-75 and DEAE-Cellulose chromatography. It was a monomeric enzyme with molecular weight of 30 kDa. The optimum pH, temperature, and NaCl for maximum protease activity were 13.0, 95°C, and 30%, respectively. Activity was stimulated by Ca^2+^, Mg^2+^, DTNB, *β*-mercaptoethanol, and SDS. The protease was extremely stable even at pH 13.25, 90°C, and 30% NaCl and in the presence of hydrophilic, hydrophobic solvents at high concentrations. The high compatibility with ionic, nonionic, and commercial detergents confirms the utility as an additive to cleaning products. Kinetic and thermodynamic characterization of protease revealed *K*_*m*_ = 1 mg mL^−1^,  *V*_max_ = 217.5 U mL^−1^, *K*_cat_/*K*_*m*_ = 99 mg mL^−1^ S^−1^, *E*_*a*_ = 51.5 kJ mol^−1^, and Δ*G* ^⁎^ = 56.5 kJ mol^−1^.

## 1. Introduction

Thermophilic and hyperthermophilic organisms play a key role in the recent research and the enzymes produced by these microorganisms are coveted for applications in many areas. The proteases are some of the most commercialized enzymes with over 65% of total enzyme market [1]. They are used in various catalytic applications in both food and pharmaceutical industries. Nowadays, their roles in the synthesis of bioactive peptides and as additive in commercial detergents are gaining attention [2]. To be incorporated in the formulation of detergents, the protease must be active and stable in harsh washing conditions like high temperature, alkaline pH, metal ions, and high salt concentrations in addition to stability and compatibility with surfactants and detergents [3, 4]. They are used as additives instead of other chemicals harmful to the environment. Since the first alkaline protease Carlsberg produced by* Bacillus licheniformis* was commercialized as an additive to detergents in the sixties [5], research has emphasized trying and characterizing new proteases with better performance. Several proteases have been purified and characterized from* Bacillus circulans* DZ100 [6],* Streptomyces *sp. AB1 [7],* Bacillus subtilis* AP-MSU6 [8],* Bacillus* sp. EMB9 [9], and* Geobacillus caldoproteolyticus* [10]. The industrial demand of highly active and stable proteases continues to rise and various attempts have been made to enhance stability of alkaline proteases by site directed mutagenesis and protein engineering but the screening of microorganisms from extreme habitats seems to be the best approach. Also in this study a protease from* Geobacillus toebii* LBT 77 isolated from a Tunisian hot spring was purified and characterized.

## 2. Materials and Methods

### 2.1. Substrates and Chemicals

Unless otherwise specified, all substrates, chemicals, and reagents were purchased from Sigma-Aldrich (Saint Louis, Missouri, USA).

### 2.2. Microorganism

The strain* Geobacillus* sp. LBT 77 producing alkaline proteases was isolated from the hot spring “Hammam El Atrous” next to the Ichkeul lagoon in Bizerte, Tunisia (37°08′18.8′′N, 9°41′24.7′′E). The strain was identified based on the phenotypic characteristics of the* Bacillus* genus and phylogenetic analysis of the 16S rDNA sequence. Genomic DNA was extracted as described by Marmur [11] and 16S rRNA gene sequences were amplified using the bacterial universal primers 16SF (5′AGAGTTTGATCCTGGCTCAG3′) and 16SR (5′CTACGGCTACCTTGTTACGA3′) [12] using the following PCR program: 1 cycle of 94°C for 5 min, 30 cycles of 94°C for 1 min, 54°C for 1 min, and 72°C for 1.5 min, and a cycle of 72°C for 5 min. The amplified products were purified and the sequence of the 16S rRNA gene was determined by Sanger method DNA sequencing (ABI 3730xl DNA analyzer, USA). Sequence comparison with the databases was performed using BLAST program through NCBI website. A phylogenetic tree was constructed with MEGA version 6.06 using the neighbor-joining method.

### 2.3. Screening and Production of Protease Activity

Screening of protease activity was performed by the method of dissemination through wells on milk agar medium at pH 9 containing 5 g/L tryptone, 3 g/L yeast extract, 15 g/L agar, and 25 mL skimmed milk. 60 *μ*L of an 18 h bacterial culture was injected into each well and the plates were incubated for 18 h at 55°C. Protease activity was confirmed by the appearance of a clear zone around the well-testifying degradation of casein milk [6]. Production of protease by* Geobacillus toebii* LBT 77 was carried out at pH 8 in a medium containing 5 g bactopeptone, 5 g yeast extract, 5 g NaCl, 10 g gelatin, and 0.2 g CaCl_2_ in 1 L deionised water [9]. Inocula were routinely grown in Tryptic Soy Broth (Scharlau, Spain) medium. Media were autoclaved at 120°C for 20 min. Cultures were performed on a rotator shaker (150 rpm/min) for 96 h at 55°C, in 500 mL Erlenmeyer flasks with a working volume of 100 mL. Growth was followed by measuring the optical density at 600 nm every 6 h. The culture medium was centrifuged at 12000 rpm for 20 min at 4°C and the cell-free supernatant was used as a crude extract for estimation of proteolytic activity.

### 2.4. Assay of Protease Activity

Proteolytic activity was determined by using casein as substrate. One mL of casein 10 g/L in 50 mM glycine-NaOH buffer pH 11 was mixed with 900 *μ*L of glycine-NaOH. Reaction was initiated by the addition of 100 *μ*L of enzyme and the tubes were placed in a water bath at 70°C. After 20 min of incubation, 2 mL of 10% TCA was added to stop the reaction. The reaction mixture was centrifuged at 12000 rpm for 10 min and absorbance was measured at 280 nm [13]. One unit of proteolytic activity was defined as the amount of enzyme required to release 1 *μ*g of tyrosine per minute under experimental conditions.

### 2.5. Enzyme Purification

#### 2.5.1. Ammonium Sulfate Precipitation

Cell-free supernatant was collected by centrifugation at 12000 rpm for 20 min at 4°C after 72 h of cultivation. Ammonium sulfate was slowly added to the supernatant to 80% saturation and the mixture was incubated overnight at 4°C. The precipitate was collected by centrifugation, dissolved in a minimal volume of 25 mM Tris-HCl (pH 8.5), and dialyzed against three changes of the same buffer for 24 h.

#### 2.5.2. Sephadex G-75 Gel Filtration

The dialysate was subjected to gel filtration on Sephadex G-75 column (2.1 cm × 50 cm) equilibrated with 25 mM Tris-HCl (pH 8.5). The column was eluted with the same buffer at a flow rate of 20 mL/h, and fractions of 3 mL were collected and then analyzed for protease activity and protein concentration. The active fractions were pooled and subjected to ion exchange chromatography.

#### 2.5.3. DEAE-Cellulose Ion Exchange Chromatography

This round was performed by applying active fractions from the previous step to a DEAE-Cellulose (2 cm × 25 cm) equilibrated with 25 mM Tris-HCl (pH 8.5). Bounded proteins were eluted with a linear gradient of NaCl from 0 to 1 M at a flow rate of 50 mL/h and analyzed for protease activity.

All purification steps were performed at 4°C. The protein content of each chromatographic fraction was determined by measuring the absorbance at 280 nm.

### 2.6. Protein Estimation

Protein concentration was determined by the colorimetric method of Bradford [14] using bovine serum albumin as standard.

### 2.7. Polyacrylamide Gel Electrophoresis and Zymography

SDS-PAGE was carried out to determine the purity and molecular weight of the enzyme as described by Laemmli et al. [15] using 5% (w/v) stacking and 12% (w/v) separating gels. Protein bands were visualized by staining with Coomassie Brilliant Blue R-250. The molecular weight of the enzyme was estimated using a low-molecular weight calibration kit (Biomatik Co., Canada) as markers.

Zymography was performed by the method of Garcia-Carreno [16] to confirm the enzyme activity. After electrophoresis, the gel was immersed in 100 mM Tris-HCl buffer (pH 8.5) containing 2.5% Triton X-100 at 4°C, with shaking for 30 min to remove SDS; then, the gel was washed twice with 100 mM Tis-HCl buffer (pH 8.5) in order to eliminate residual Triton X-100 and then incubated with 1% (w/v) casein in 100 mM Tris-HCl (pH 9.5) at 75°C for 60 min. Finally, the gel was stained using Coomassie Brilliant Blue R-250. Appearance of a clear halo zone on the dark-blue background indicates the presence of enzyme.

### 2.8. Biochemical Properties of the Purified Protease

#### 2.8.1. Effects of Temperature, pH, and NaCl on Enzyme Activity and Stability

To study the effect of temperature on activity of the purified enzyme, the reaction mixture was incubated at different temperatures ranging from 55 to 120°C. Enzyme activity was measured as described earlier. Thermal stability was evaluated by incubating the purified enzyme at 70, 80, 90, and 95°C for 180 min in the presence and absence of 5 mM CaCl_2_. Residual activity was measured at 95°C and pH 13. The activity of nonheated enzyme was considered as 100%.

Effect of pH was determined by varying the pH of the reaction mixture using the following buffers: (100 mM) glycine-HCl, pH 2.0–5.0; potassium phosphate, pH 6.0–7.0; Tris-HCl, pH 8.0–8.5; glycine-NaOH, pH 9.0; NaHCO_3_-NaOH, pH 9.5–10–11; NaH_2_PO_4_-NaOH, pH 11.5–12; KCl-NaOH, pH 12,5–13–13.5–13.75. To test pH stability, the enzyme was preincubated in various buffer solutions (pH 6–13) for 12 h at 60°C. The residual enzyme activity was then determined at the optimum conditions of assay.

Effect of NaCl was carried out by incubating the reaction mixture at different concentrations of NaCl (0–40%). Stability was investigated by preincubating the enzyme for 1 h at different concentrations of NaCl (0–40%) and residual activity (%) was measured at the optimum conditions of assay.wa

#### 2.8.2. Effects of Inhibitors and Metallic Ions on Activity of Protease

Effect of protease inhibitors such as phenylmethylsulfonyl fluoride (PMSF), ethylenediaminetetraacetic acid (EDTA), dithio-bis-nitrobenzoic acid (DTNB), and *β*-mercaptoethanol was determined at 5 and 10 mM by measuring relative activity after preincubating the enzyme for 30 min at 55°C. The activity at standard conditions without additives was considered as 100% [2]. Effect of metal ions on enzyme activity was investigated at 5 mM by adding divalent (Zn^2+^, Fe^2+^, Mg^2+^, Mn^2+^, Ca^2+^, Cu^2+^, Hg^2+^, and Co^2+^) and monovalent (Na^+^, K^+^, Al^+^, and Li^+^) ions to the reaction mixture. Relative activities were estimated versus activity without any metallic ions after 1 h of incubation at 55°C.

#### 2.8.3. Effect of Detergents on Enzyme Activity

Anionic (SDS), cationic (CTAB), and nonionic detergent (Triton X100) were preincubated with enzyme for 60 min at 55°C and residual activity was determined. Effect of commercial detergent (OMO, Ariel, and Nadhif) on protease activity was studied with 1% (w/v) solution of detergent. The enzyme was preincubated with the aforementioned detergents at 50°C for 1 h and then assayed for protease activity.

#### 2.8.4. Effect of Organic Solvents on Protease Activity

The enzyme was mixed with 25 and 50% of many organic solvents such as cyclohexane, n-butanol, ethanol, toluene, acetonitrile, acetone, isopropanol, methanol, and benzene for 4 h at 55°C.

### 2.9. Catalytic and Thermodynamic Parameters

Kinetic parameters were determined by assaying protease activity against various casein concentrations under optimized assay conditions. *V*_max_, *K*_*m*_, *K*_cat_, and *K*_cat_/*K*_*m*_ were calculated using a Lineweaver-Burk plot (1/*V* versus 1/[*S*]). Thermodynamic parameters for casein hydrolysis, activation energy, enthalpy, entropy, and free energy of activation were calculated as per Akolkar and Desai [17].

All experiments mentioned above were repeated at least three times, and each value represents the average of three repetitions.

## 3. Results and Discussion

### 3.1. Microorganism

**Figure 1:**
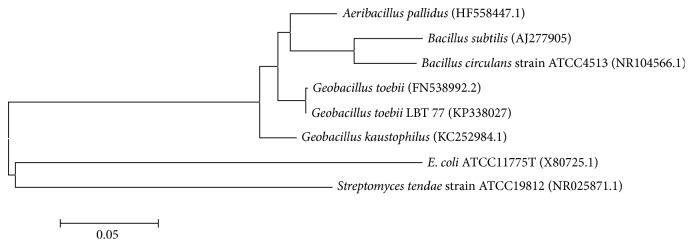
Phylogenetic tree based on 16S rRNA gene sequences, drawn using the neighbor-joining method and showing the relationship between* Geobacillus toebii* LBT 77 and species from genera* Bacillus* and* Geobacillus*.* E. coli* and* Streptomyces tendae* were used as out groups.

From a collection of 161 bacteria, 37 caseinolytic strains were isolated. Among these strains, the strain LBT 77 exhibited a large clear zone of degradation showing high protease activity. Phylogenetic analysis of its 16S rRNA gene sequence indicated that the strain LBT 77 is affiliated to* Geobacillus* and is closest to* Geobacillus toebii* (99% homology) (Figure 1). The sequence has been deposited in the EMBL/GenBank/DDBJ databases under accession number KP338027.

### 3.2. Production of Extracellular Protease

**Figure 2:**
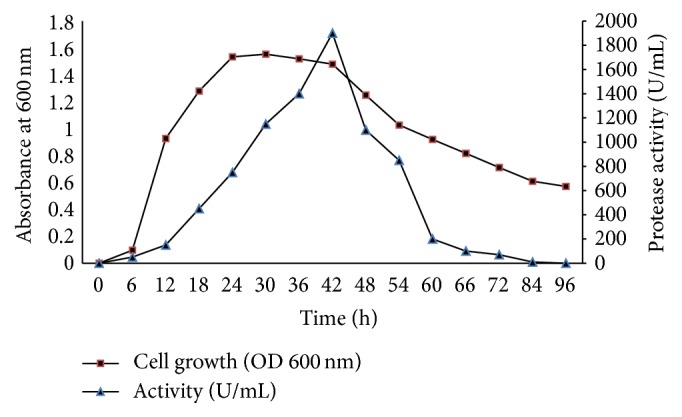
Growth kinetics and protease production of* Geobacillus toebii* LBT 77.

Protease production is proportional to bacterial growth; in fact, the maximum production coincides with the end of the stationary phase (1900 U/mL) at 42 h of incubation (Figure 2). This result is consistent with reports in the literature [2, 18, 19].

### 3.3. Purification of Protease

**Figure 3:**
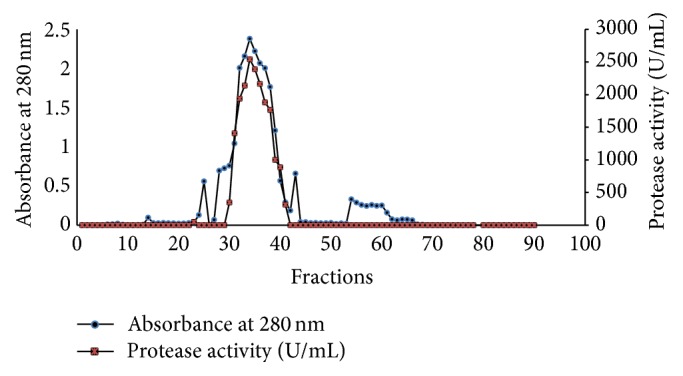
Elution profile of protease from* Geobacillus toebii* LBT 77 on Sephadex G-75 gel filtration chromatography.

The protease present in the crude extract was purified using ammonium sulfate 80% followed by Sephadex G-75 and DEAE-Cellulose (Figure 3).  Table 1 indicated that the protease was purified to 5-fold with specific activity of 739.5 U/mg. The reduction of recovery (%) from one step to the other is due to the elimination of some lower specific activity during chromatography as reported previously [20].

### 3.4. Polyacrylamide Gel Electrophoresis and Zymography

**Figure 4:**
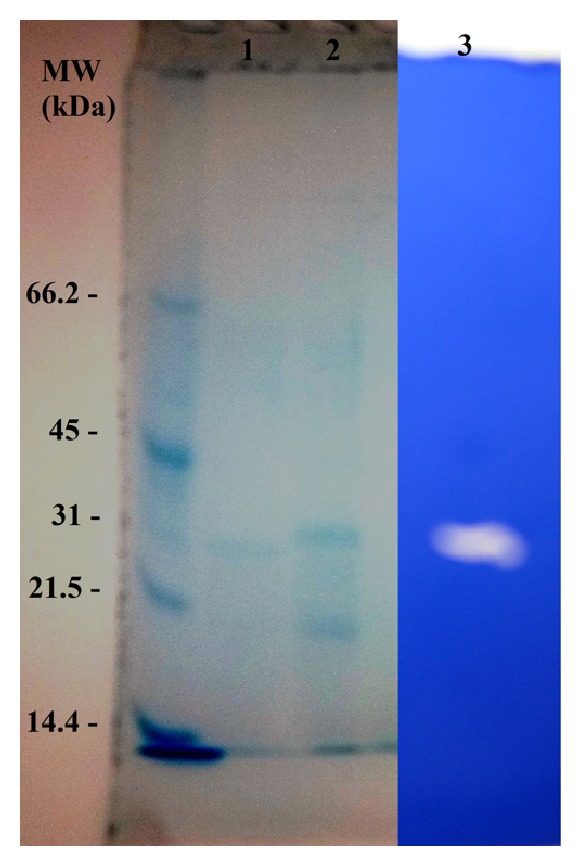
SDS-PAGE and activity staining of purified* G. toebii* LBT 77 protease. Lane 1: purified proteases after DEAE-Cellulose; Lane 2: proteases purified by sephadex G75; Lane 3: zymogram of purified protease.

The purified protease appeared as a single band on the SDS-PAGE with a molecular weight of approximately 30 kDa (Figure 4). Mostly, the molecular weights of bacterial proteases are between 15 and 40 kDa. The molecular weight of the protease produced by LBT 77 was lower than those of the* Bacillus pumilus* CBS (34,5 kDa) [21] and* Bacillus circulans* DZ100 (32 kDa) [6] and higher than those of the proteases from* Bacillus mojavensis* A21 (20 kDa) [22] and* Bacillus subtilis* PE11 (15 kDa) [23].

The zymogram analysis showed a clear band against a blue background indicating the purity and the monomeric form of the protease.

### 3.5. Biochemical Properties of the Purified Protease

#### 3.5.1. Effects of Temperature, pH, and NaCl on Enzyme Activity and Stability

**Figure 5:**
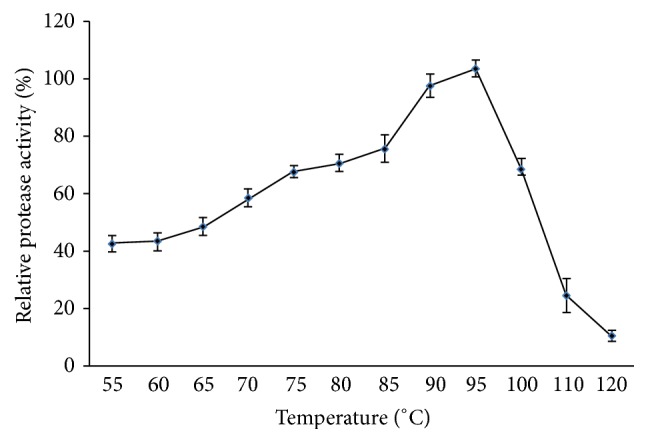
Effect of temperature on protease activity.

The enzyme was active between 70 and 100°C with an optimum of 95°C (Figure 5). Relative activities at 85, 90, and 100°C were about 72, 94, and 65%, respectively. An optimum of temperature at 95°C was reported for the protease from* Bacillus* sp. MLA64 [24].

**Figure 6:**
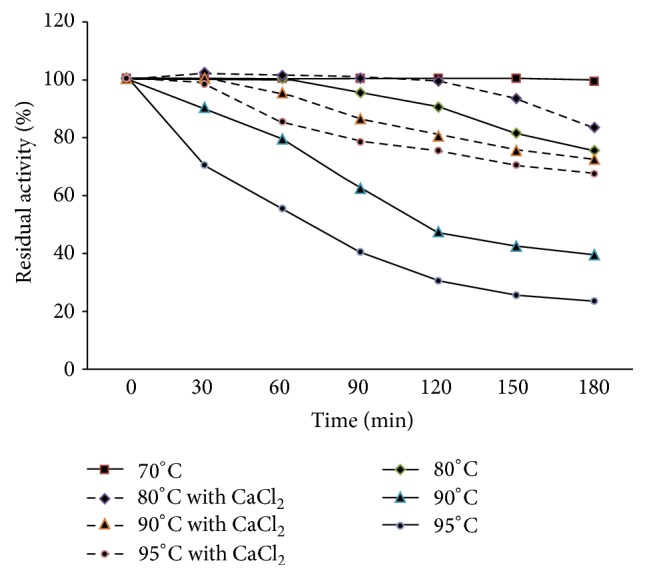
Effect of temperature on protease stability.

The protease in this study was also found to be completely stable at temperatures lower than 80°C after 180 min of incubation (Figure 6). Half-life of the enzyme at 95°C was estimated to be 70 min. This is higher than those of other proteases which retain a lower amount of their initial activity even in a shorter period of incubation and also at lower temperatures. Ca^2+^ increased thermal stability of the enzyme since it retained 67% of the original activity after incubation at 95°C for 180 min. The effects of Ca^2+^ on thermal stability of proteases were previously reported [25].

**Figure 7:**
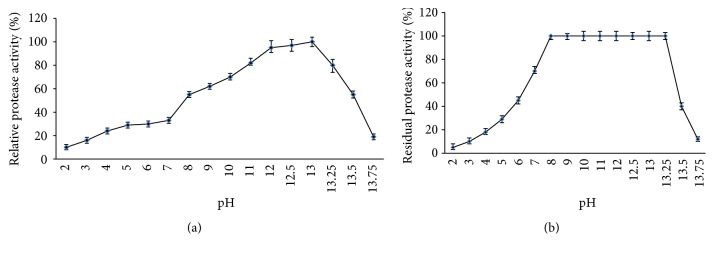
Effect of pH on protease activity (a) and stability (b).

The enzyme showed maximum activity at pH 13.0 and was highly active in the pH range of 9.0 to 13.25. Indeed, the enzyme shows 62% and 80% of its activity at pH 9 and 13.25, respectively (Figure 7(a)). These findings indicate that this enzyme belonged to alkaline proteases group. The particularity of this enzyme is its pH optimum of activity. In fact, most alkaline proteases listed in the bibliography have optimum pH that does not exceed pH 12 [26, 27]. The enzyme was stable between pH 8 and 13 and retained about 99% of its activity after incubation at 50°C for 12 h (Figure 7(b)). These characteristics are important for its use as laundry additive [28].

**Figure 8:**
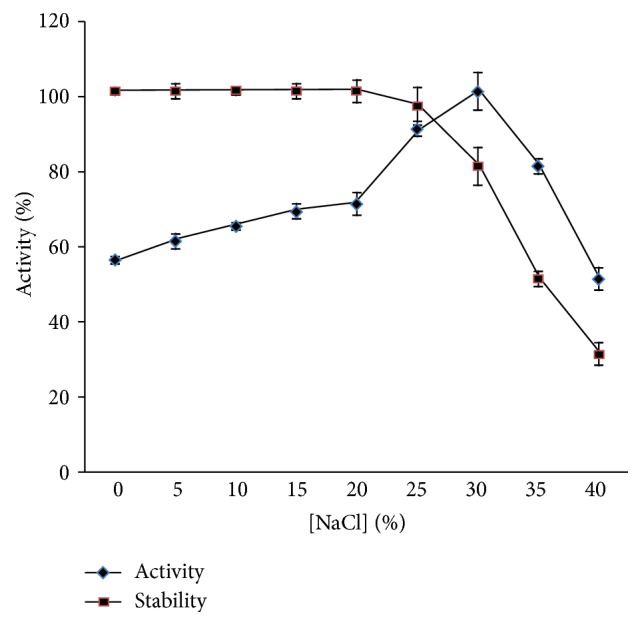
Effect of [NaCl] on protease activity and stability.

The protease activity has risen by increasing NaCl concentration until an optimum of 30% and has decreased at higher concentration. The same optimum concentration was observed for the protease produced by* Bacillus alveayuensis* CAS 5 [29]. Halotolerant proteases are interesting for biotechnological applications, especially in detergent industry, and this one was completely stable at 20% and retained 80% of activity at 30% of NaCl (Figure 8). This was higher than the protease of* Bacillus aquimaris* VITP 4 [19] which was stable up to 12% and lower than this of* Bacillus alveayuensis* CAS 5 [29] which was stable up to 25%.

#### 3.5.2. Effect of Inhibitors and Metal Ions on Activity of Protease

**Figure 9:**
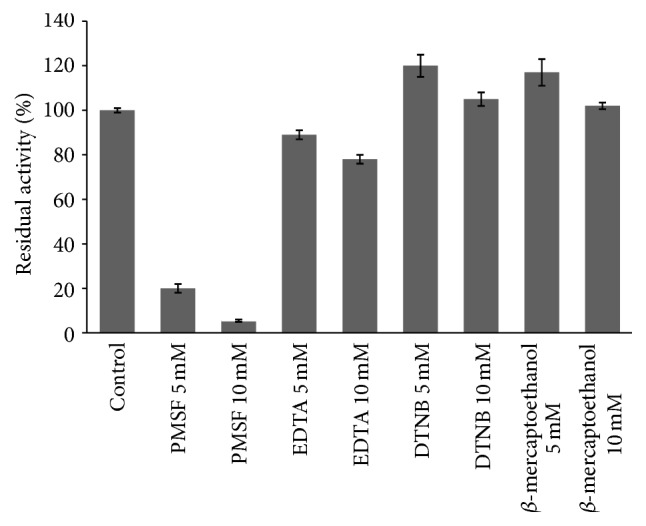
Effect of protease inhibitors on enzyme activity.

**Figure 10:**
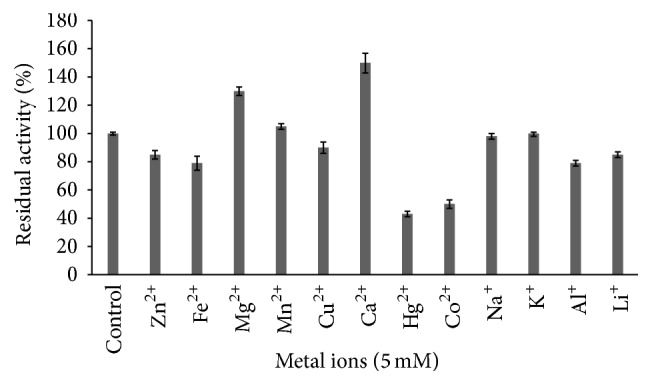
Effect of monovalent and bivalent ions on protease activity.

The effect of inhibitors and chelators on proteases activity showed that PMSF strongly inhibits the activity to 20% and 5% at 5 and 10 mM, respectively (Figure 9). This confirms its belonging to the group of serine proteases [25, 30]. Among the tested inhibitors, activity was increased by DTNB and *β*-mercaptoethanol suggesting that it is a thiol-depending serine protease [25]. The metalloproteases inhibitor EDTA inhibited activity by 22% at 10 mM; similar result was reported earlier in serine protease produced by* Bacillus licheniformis* [31]. Regarding the effect of metal ions, the activity was enhanced with Mg^2+^ and Ca^2+^ (Figure 10). This was similar to result of protease from* Geobacillus* sp. YMTC 1049 [32]. It has been reported that serine proteases, such as subtilisin, have Ca^2+^ binding sites, and their stability at higher temperatures was explained by the strengthening of interactions inside protein molecules and the better stabilization of active site against thermal denaturation [33].

The enzyme was quite stable with Mn^2+^, K^+^, Na^+^, Zn^2+^, Fe^2+^, Cu^2+^, Al^+^, and Li^+^ at 5 Mm. The repressive effect of Zn^2+^ and Cu^2+^ was reported earlier [2, 34]. Metals like Hg^2+^ and Co^2+^ strongly inhibit the activity compared to control.

#### 3.5.3. Effect of Detergents on Enzyme Activity

**Figure 11:**
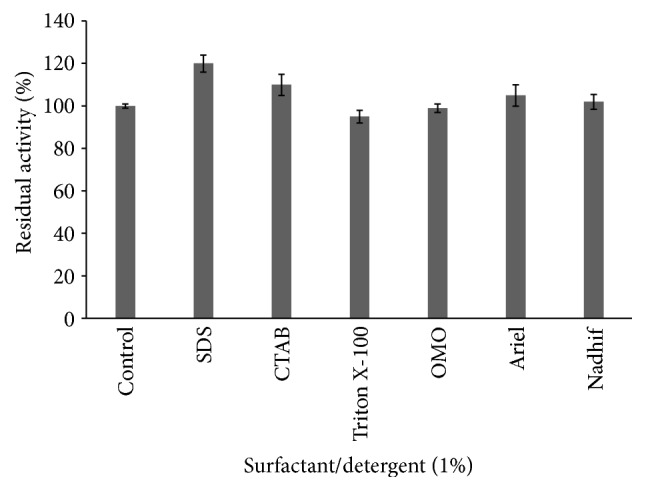
Effect of surfactants and detergents on protease activity.

All tested detergents did not inhibit protease activity but rather an enhancement was observed with SDS and CTAB (increasing activity by 20 and 10%, resp.). This result is consistent with those reported for alkaline proteases from* Bacillus* sp. and* Bacillus clausii* [35, 36]. In addition, the protease produced by* Geobacillus toebii* LBT 77 was also compatible with commercial detergents like OMO, Nadhif, and Ariel (Figure 11).

#### 3.5.4. Effect of Organic Solvents on Protease Stability

**Figure 12:**
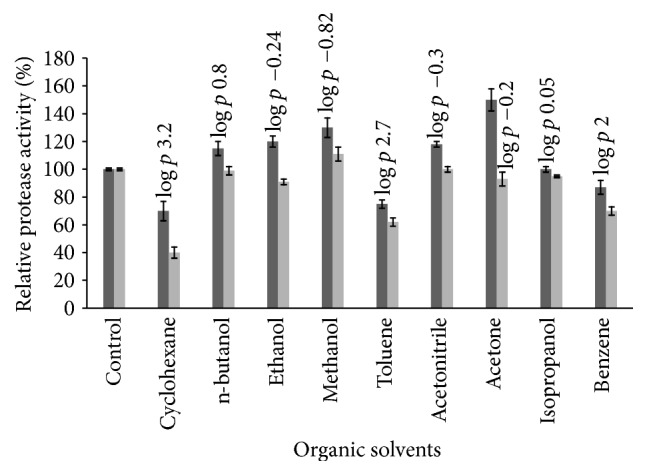
Effect of organic solvents on protease activity.

The effect of various organic solvents at 25 and 50% (v/v) on protease stability is shown in Figure 12. Protease activity was increased in the presence of acetonitrile, methanol, ethanol, and n-butanol at 25% v/v. A similar result was reported for the protease produced by* Bacillus* sp. EMB9 [9]. The most interesting observation is that the activity remains stable with acetone (93% of activity) unlike protease from* Bacillus* sp. EMB9 which retains only 20% of its initial activity [9]. Activity was stable with isopropanol, acetonitrile, and n-butanol at 50% v/v. There is a small decrease in activity in the presence of 50% ethanol (91% of activity).

The enzyme keeps 70, 62, and 40% of its activity in the presence of 50% benzene, toluene, and cyclohexane, respectively. Protease from* Geobacillus toebii* LBT 77 was found stable in solvents concentration 50% even after 4 h at 55°C. This was found to be superior stability compared to protease characterized from* Bacillus* sp. and* Bacillus* sp. EMB9 which retain only about 20% of their activity [2, 9].

### 3.6. Catalytic and Thermodynamic Parameters

Thermodynamic and kinetic parameters shown in Table 2 confirm the high interest of the enzyme. Actually, catalytic efficiency of the serine protease from LBT 77 (99 mg mL^−1^ S^−1^) is better than those of* Bacillus pumilis* CBS [21] and* Halobacterium* sp1(1) [17] with 45 and 85 mg mL^−1^ S^−1^, respectively.

Briefly, the present study demonstrated that the purified serine protease from the thermophilic* Geobacillus toebii* strain LBT 77 has a number of properties that make it a promising potential candidate for application in the detergent industry as a bioadditive in detergent formulation. In fact, it showed high levels of thermoactivity and thermostability and a marked stability to detergents. The enzyme also exhibited high levels of stability against pH, NaCl, ions, detergents, and solvent which responds to the industrial requirements.

## Figures and Tables

**Table 1:** Purification of protease from *Geobacillus toebii* LBT 77.

Purification step	Total activity (U)	Total protein (mg)	Specific activity (U/mg)	Recovery (%)	Purification fold
Crude enzyme	11357	171.5	66.5	100	1
Ammonium sulfate precipitation	8064	45	180	71	1.5
Sephadex G-75	2504	7	348	22	4.5
DEAE-Cellulose	2292.5	3	739.5	20	5

**Table 2:** Kinetic and thermodynamic parameters of the purified enzyme.

Kinetic parameters		Thermodynamic parameters	
*K*_*m*_ (mg/mL)	1	*E*_*a*_ (kJ mol^−1^)	51.5
*V*_max_ (U/mL)	217.5	Δ*H* ^*∗*^ (kJ mol^−1^)	5
*K*_cat_ (S^−1^)	94.5	Δ*S* ^*∗*^ (J mol^−1^)	−229
*K*_cat_/*K*_*m*_(mg mL^−1^ S^−1^)	99	Δ*G* ^*∗*^ (kJ mol^−1^)	56.5
